# Socioeconomic Status and Ovarian Cancer Stage at Diagnosis: A Study Nested Within UKCTOCS

**DOI:** 10.3390/diagnostics10020089

**Published:** 2020-02-07

**Authors:** Chloe Karpinskyj, Matthew Burnell, Arturo Gonzalez-Izquierdo, Andy Ryan, Jatinderpal Kalsi, Ian Jacobs, Max Parmar, Usha Menon, Aleksandra Gentry-Maharaj

**Affiliations:** 1 MRC CTU at UCL, Institute of Clinical Trials and Methodology, University College London, 90 High Holborn, London WC1V 6LJ, UK; c.karpinskyj@ucl.ac.uk (C.K.); m.burnell@ucl.ac.uk (M.B.); a.ryan@ucl.ac.uk (A.R.); a.gentry-maharaj@ucl.ac.uk (A.G.-M.); 2 Institute of Health Informatics, University College London, 222 Euston Road, London NW1 2DA, UK; arturo.gonzalez-izquierdo@ucl.ac.uk; 3 Institute for Women’s Health, University College London, 84-86 Chenies Mews, London WC1E 6HU, UK; j.k.kalsi@ucl.ac.uk (J.K.); i.jacobs@unsw.edu.au (I.J.); 4 University of New South Wales, Sydney 2052, Australia

**Keywords:** tubo-ovarian cancer, socioeconomic status, SES, stage, deprivation, epidemiology, ovarian cancer, IMD

## Abstract

Background: Tubo-ovarian cancer (OC) continues to be the most lethal of all gynaecological cancers. Over half of women are diagnosed with late stage (III/IV) disease, which has a five-year survival rate of 11%. Socioeconomic status (SES) has been shown to have an impact on outcomes of several cancer types, including OC. This study aims to investigate any potential association between SES and stage at diagnosis of OC. Methods: Women from the non-screening arm of the United Kingdom Collaborative Trial of Ovarian Cancer Screening (UKCTOCS) with a confirmed diagnosis of OC prior to 01 January 2015 and an English index of multiple deprivation (IMD) score were eligible for the study. The association between IMD and OC stage (FIGO) was analysed using an ordinal logistic regression model adjusted for age at diagnosis and BMI. Results: Four-hundred and fifty seven women were eligible for inclusion in the primary analysis. The odds of being diagnosed with the higher dichotomization of stage (I vs. II/III/IV; I/II vs. III/IV; I/II/III vs. IV) was 1.29 (*p* = 0.017; 95% CI: 1.048–1.592) per unit SD (standard deviation) increase in IMD. This translates to a 29% increase in odds of being diagnosed at the higher stage per each unit SD increase in IMD. Conclusion: Increased deprivation is consistently associated with a higher probability of being diagnosed with later stage OC.

## 1. Introduction

Despite advances in treatment over the past three decades, tubo-ovarian cancer (OC) remains the most lethal of all the gynaecological cancers [1]. Most women are diagnosed with late (III-IV) stage disease, which has five-year survival rates of around 11% [2]. However, survival for women diagnosed at stage I and II is over 90% and 68%, respectively. This suggests that any improvements in detecting the disease at an earlier stage is likely to have an impact on survival [3]. In addition, increasing the proportion of women diagnosed with microscopic peritoneal disease (Stage IIIA) who are amenable to complete surgical resection will also improve survival [4]. 

Socioeconomic status (SES) describes the intersection of an individual’s income, education and occupation. Most studies have investigated the impact of SES on cancer survival, with few focussing on stage. On the most part, higher SES is associated with better survival, but it is as yet unclear whether this is a result of earlier diagnosis, improved treatment or a combination of both. 

Lower SES has been shown to be associated with an increased likelihood of late- stage diagnosis in a number of cancers, such as breast [5], colorectal [6] and pancreas [7]. However, there have been fewer studies and no consensus drawn with regards to OC. To date, six studies have investigated the association between SES and OC stage at diagnosis (early vs. late) (Table 1). A US population-based study found an association between greater individual affluence and earlier stage at diagnosis, but not area-level disadvantage [8]. This is in keeping with a later English population-based study spanning 2006-10, where a non-statistically significant trend (*p* = 0.077) was reported between Index of Multiple Deprivation (IMD, the UK government’s official area-level measure of deprivation) and late stage (III/IV) disease [9]. The largest study to date (*n* = 16,228), which used seven SES indicators from Census data to calculate an area-level index score found no evidence of any association between this measure and OC stage, however it did report a difference according to individual health insurance status [10]. In a population-based Scottish study, there was a borderline/weak association but it was unclear how deprivation was determined [11]. Interestingly, two recent studies have both reported increased risk of advanced disease at diagnosis in women with lower educational attainment [12,13].

In the multi-centre UK Collaborative Trial of Ovarian Cancer Screening (UKCTOCS), we had complete data on a population-based series of tubo-ovarian cancer cases diagnosed between 2001 and 2014, where cancer site and stage had been independently confirmed. We report on the association between stage at diagnosis of OC and SES in clinically presenting women in the ‘no screening’ arm of the trial. 

## 2. Materials and Methods 

UKCTOCS is a population-based randomised controlled trial (RCT) designed to assess the effectiveness of two screening strategies in reducing ovarian cancer mortality. As described elsewhere [14], to be eligible, women had to be aged 50–74, postmenopausal and have no family history of ovarian cancer. Invites were sent out to over 1.2 million potentially eligible women to take part in the trial (one in six of the entire eligible pool in the UK). From this, 202,638 population-risk women at 13 regional centres (RCs) across England (*n* = 10), Wales (*n* = 2) and Northern Ireland (*n* = 1) were randomised to annual screening using either ultrasound, CA125 blood test (interpreted using the Risk of Ovarian Cancer Algorithm, ROCA), or no screening (control), in a 1:1:2 fashion. The women who subsequently developed ovarian cancer represented an unselected population series of women with the disease. UKCTOCS was approved by North West—aydock Research Ethics Committee in 2000 (ref 00/8/034), and this analysis was approved as Substantial Amendment on 24 January 2017.

### 2.1. Identification of Cases

Tubo-ovarian cancer cases were identified via electronic health record linkage using NHS number to national registries and two postal follow-up questionnaires. Data were obtained for this analysis on the following dates—England and Wales cancer and mortality registry data from NHS Digital, July 2018; NI death registry data from Northern Ireland Health and Social Care Business Service Organisation, May 2018; English Hospital Episode Statistics (HES), March 2018; and Welsh hospital episode statistics (PEDW), December 2017. Two postal follow-up questionnaires were sent; the first 3–5 years following randomisation (FUQ1) and the second in 2014 (FUQ2). 

Health records detailing presentation, diagnosis and treatment were obtained whenever one of 19 pre-specified ICD-10 (International Classification of Disease) codes were reported (which included ovarian cancer, C56; fallopian tube cancer, C57; primary peritoneal cancer, C48.1/C48.2; and C80, cancer of unspecified site) [15] in electronic health records or when the volunteer indicated an OC diagnosis in afollow up questionnaire (FUQ). Records were obtained from primary, secondary and tertiary care providers and reviewed by an independent outcomes review committee consisting of gynaecological oncologists and pathologists who were blinded to randomisation group. They assigned site, stage (FIGO) and Type I/II classification, the latter based on histological subtype and grade [16].

As screening aims to intervene in disease detection, cases in this analysis were restricted to women diagnosed in the ‘no screening’ or control arm, to be representative of the clinical pathway. All women with a diagnosis date up to 31 December 2014 were included in the analysis.

The primary outcome was defined as invasive epithelial tubo-ovarian cancer as per WHO (World Health Organisation) classification 2014 [17]. Of note, this classification includes the majority of the cancers that were classified under the WHO 2013 classification as primary peritoneal cancer. Stage was defined as per FIGO 1988 [18].

### 2.2. Determination of SES

IMD (Index of Multiple Deprivation) was used to measure SES due to the precision afforded by the relatively few people per area (~1500 people). Analysis was restricted to women resident in England, as the equivalent Wales and Northern Ireland datasets were not commensurate. IMD divides England into 32,844 small areas and ranks them from least to most deprived, based on a combined score made up of 38 indicators across seven weighted domains (income; employment; education, skills and training; health and disability; crime; barriers to housing and services; and living environment). Residential postcode was collected at recruitment and updated by the volunteer or from health records where available. It was mapped to IMD using the most recent address on record. The 2010 IMD dataset was used, as it was contemporaneous with the diagnosis date of the majority of eligible participants (median, June 2009; IQR Nov 2006–June 2012). Self-reported individual-level measures of SES (highest level of education attained; none, ‘O’ level, ‘A’ level, clerical/commercial qualification, nursing/teaching qualification, and college university qualification) were available for those who responded to FUQ1. They were included in the complementary analysis.

### 2.3. Identification of Covariates

At recruitment, women provided date of birth, height, weight, ethnicity and information related to their reproductive health/history. At FUQ1, women provided smoking status (ever, packs per day and number of years smoked), units of alcohol consumed per week, highest level of educational attainment and other medical history. Due to the small proportion of non-White participants (2.4%), ethnicity was not included as a covariate. Of note, England and Wales census data from 2011 reports that 93% of women aged between 50 and 74 are of White ethnicity [19], slightly lower than in our study (97.6%).

### 2.4. Statistical Analysis

The primary analysis was restricted to cases where there was no missing data on the English IMD score, age at diagnosis and BMI. IMD score was kept as continuous for reasons of interpretation and statistical power. IMD was also standardised relative to the English population, so that the regression parameter could be interpreted as the effect of an increase by one standard deviation of deprivation score in England. 

An ordinal logistic regression model was used to regress stage at diagnosis on IMD score, plus age at diagnosis and BMI. Fractional polynomials were used to determine the most appropriate functional form for IMD (and age at diagnosis) in the model. A likelihood ratio test (LRT) comparing this model against a generalised ordinal logistic regression model assessed the proportional odds assumption across the outcome categories (stages I to IV). A random effects ordinal logistic regression model was fitted to establish whether there was any unexplained heterogeneity across regional centres that needed to be accounted for. 

To aid interpretability of the model, the marginal effect on the probability of each stage for a range of IMD scores was plotted with 95% confidence bands. This was also repeated with the probabilities stacked so that cumulative effect over stages I to IV could be visualised. 

A complementary analysis was undertaken on those with additional data from FUQ1 on educational attainment level, ever smoking and alcohol consumption. 

All analyses were performed using Stata version 15.1, including the user-written command gologit2 [20]. 

## 3. Results

Of the 101,299 women randomised to the control arm, 679 were confirmed by the outcomes review committee to have invasive tubo-ovarian cancer diagnosed between 2001 and 2014. Of the 679 women, 477 were resident in England. Twenty-one women had to be excluded from the analysis due to inability to link to an English IMD score due to incorrect postcode (15), lack of valid BMI (5), or missing stage (1). Complete covariate data for the primary analysis model was available in 457 (95.8%, 457/477). The complementary analysis, which included co-variate data from FUQ1 on smoking, alcohol and education was limited to 265 cases (Table 2). 

### 3.1. Baseline Characteristics

The median age at diagnosis of OC of the 457 women included in the primary analysis was 68.4 (IQR 63.18–73.82). Overall, 23.0% (105/457) of women were diagnosed with early stage (I/II) and 77.0% (352/457) with late stage (III/IV) disease. The majority (345/457; 75.5%) were Type II cancers (Table 3). The median BMI (25.1; IQR 22.98–28.93) was at the lower boundary of the ‘overweight’ category (BMI ≥25 and < 30 gm/M^2^) as designated by the WHO [21]. Just below half (45.1%; 206/457) of the women had a BMI in the ‘normal’ range. Of the 278 FUQ1 responders, most women reported that they consumed fewer than six units of alcohol per week (76.3%; 212/278), with 24.5% (68/278) being abstinent (Table 3).

### 3.2. Ordinal Logistic Regression

Initial use of a generalized ordinal logistic regression model indicated that the proportional odds assumption held (*p* = 0.967 when comparing models with a LRT) and the more parsimonious model could be used. Fractional polynomials (FPs) of degree 1 in the logit scale suggested that a linear function for IMD was the best fitting, whilst for FPs of degree 2, terms of power −0.5 and 2 proved the best fitting, though not a statistical improvement over the linear function (*p* = 0.912). Hence, the linear representation of IMD was retained in the model. Similarly, age at diagnosis did not have a statistically superior nonlinear functional form in the logit scale (*p* = 0.435). A further simplification was the redundancy of a random intercept term for the 10 English RCs (*p* ≈ 1). 

**Figure 1:**
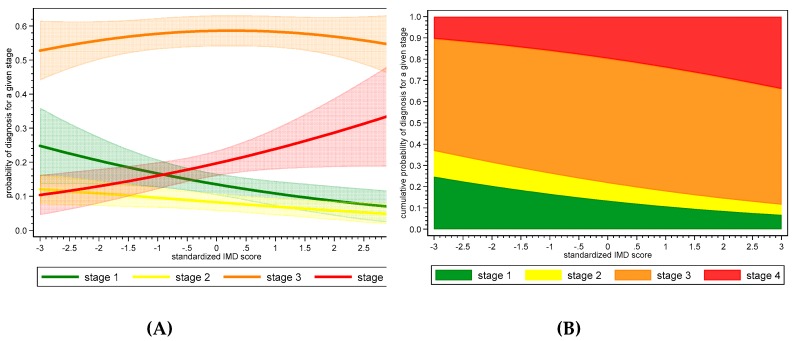
Predicted probabilities of stage of diagnosis by standardized index of multiple deprivation (IMD) score. Panel **A** includes 95% confidence bands, and Panel **B** has probabilities stacked in a cumulative manner to aid interpretation of stages in combination, (I–III, for example).

The primary analysis model, based on 457 cases and adjusted for BMI and age at diagnosis, estimated an odds ratio of 1.29 (*p* = 0.017; 95% CI: 1.048–1.592) (Table 4). Thus, a unit SD (standard deviation) increase in the deprivation level in the English population resulted in a 29% increase in the odds of the stage at diagnosis being at a higher dichotomization of stage (that is, stage I versus II,III,IV; or stage I,II versus III,IV; or stage I,II,III versus IV). Figure 1 helps visualize the impact of this OR and the three cut-point parameters on marginal estimates of the predicted probability of each stage given an IMD value. Regardless of IMD, the probability of diagnosis at stage III is over 50%, with slightly lower probabilities at very low and high IMD scores (Figure 1). However, the probability of diagnosis at either stage I or II consistently declines as IMD increases. Early stage diagnosis (I or II) declines from 27.6% for the lowest deprived decile to 16.8% for the most deprived decile. Conversely, the probability of a stage IV diagnosis increases with IMD, from 15.1% for the lowest deprived decile to 25.2% for the most deprived decile (Figure 1). 

The second, complementary analysis, including education, smoking and alcohol, produced a similar estimate of OR = 1.34 (*p* = 0.054; 95% CI: 0.994–1.805). However, due to the reduced sample size, evidence of statistical significance was marginal at conventional levels of testing (Table 2).

## 4. Discussion

Analysis of this population-based cohort of 457 independently confirmed primary invasive epithelial tubo-ovarian cancer cases demonstrates that the odds of being diagnosed with higher stage cancer consistently increases with deprivation. Per unit SD increase in IMD, the odds of higher stage disease diagnosis increases by almost a third (dichotomised as stage I versus II, III, IV; stage I, II versus III, IV; stage I, II, III versus IV).

In the literature, SES is estimated variously, using educational attainment, area-level deprivation indices and personal affluence. As SES is complex and multifactorial, there is no single ‘best’ measure [22,23]. Given this, concordant results between studies should not necessarily be expected. Three of the six published studies on SES and OC stage at diagnosis have reported strong evidence of an association between lower SES and increased odds of diagnosis with late stage disease, with one reporting weak association and two reporting none. In this study, IMD, a multidomain area-based measure based on residential postcode has been used. A 2013 English study of 2744 women with OC also used IMD. It reported no evidence of an association between deprivation and stage at diagnosis Two US studies also used composite indices similar to IMD. One reported a null result, and the other reported no difference according to neighbourhood disadvantage, but a small difference in prevalence according to neighbourhood affluence.

In our study, we explored the association of SES with invasive epithelial tubo-ovarian cancer as defined by WHO 2014, which includes invasive tubal cancer and most cases previously classified as primary peritoneal cancer [17]. This definition and analysis is in-keeping with the published literature where the strongest evidence of an association between SES and OC stage is where the analysis is limited to invasive cancers [8,12]. Other studies have limited their definition of OC to the ICD-10 code of C56 (malignant neoplasm of the ovary) and predominantly rely on use of cancer registrations as the sole source of ascertainment of OC (such as the English and US studies above). However, the ICD-10 code C56 includes noninvasive, borderline epithelial and non-epithelial ovarian tumours where the natural history results in diagnosis at an early stage. Additionally, C56 excludes tubal (ICD-10 C57) and primary peritoneal cancer (ICD-10 48.1).

We provide information regarding deprivation and probability of diagnosis for all four cancer stages. In all previous studies, stage was analysed by dividing cases into early (I and II) vs. late (III and IV), leading to a loss of information in comparison to our study. 

Due to the highly aggressive nature of the most common OC subtype (high-grade serous), there may be limited scope for interventions to improve socioeconomic equality of OC stage at diagnosis. This is compounded by reports that 30% of women over 45 experience at least one OC symptom on a frequent basis [24], due to their nonspecific nature. Furthermore, three-quarters of women with OC having advanced disease by the time they are symptomatic [25], and that foreknowledge of OC symptoms has not been shown to improve survival [26]. Underlying reasons for lower SES women delaying presentation to their primary care physician or general practitioner include higher prevalence of fatalist attitudes towards cancer and lower awareness of cancer symptoms [27]. Both of these could be addressed with targeted awareness campaigns. Preliminary results from a UK pilot study to increase awareness of OC symptoms reported an overall increase in women over 50 attending their general practitioner (GP) with persistent bloating, but no increase in those in most deprived areas [28]. This awareness campaign also resulted in only an additional 0.04 GP attendances per practice per week. To reduce inequality, implementing a lower threshold of referral for women from deprived areas who present to primary care with OC alert symptoms [29] may help to take into account patient-delay.

### 4.1. Strengths

The key strength of this study is the significant efforts undertaken to minimise selection bias. Complete identification of cases in this cohort was ensured through use of the NHS number to link to multiple electronic health records (cancer and death registries and HES) with updates until 4 years post censorship date at end 2014 and use of additional data sources such as follow-up questionnaires and reporting by trial centres. This was further augmented by a protocol that mandated retrieval of health records for outcomes review of all women diagnosed with one of 16 ICD-10 codes [15], which could potentially indicate a potential OC diagnosis, in addition to the OC specific codes (C56/57/48). Cases were confirmed by a review of healthcare records by an independent outcomes review committee of clinical experts, resulting in high-quality and complete data with regard to cancer site, stage and morphology data. In contrast, previous studies have reported a high proportion of missing stage data (~10%) [9,11]. Furthermore, IMD, a small-area composite measure and the UK government’s measure of relative deprivation, was used to determine SES. 

### 4.2. Limitations 

The cohort included women who had lower deprivation, as reflected by lowerr rates of smoking and obesity than among the age-matched UK population [30,31]. This accentuates our finding, for it suggests that the true effect size is likely to be larger. In-keeping with a less deprived population, our women had a lower average BMI and were less deprived than the general population [3]. Non-White ethnicity has previously been shown to adversely affect stage at diagnosis [32]. However, the high proportion of White women in the cohort (97.6%, ~5% higher than reported in England and Wales 2011 census data [19]) meant that it was not possible to investigate this. We were not able to include marital status, which has been shown to be associated with better outcomes in previous studies [33,34]. 

## Figures and Tables

**Table 1:** Review of current literature on SES and OC stage at diagnosis

Author	Country	Diagnosis Date of Cohort	Study Design	*n* =	Definition of OC	Indicator of SES	Covariates	Limitations	Findings	OR (95% CI)	*p*-Value
Peterson et al. [8]	USA	1994–1998	Case series	581	Epithelial ovarian cancer (including borderline tumours) from cancer registries. A proportation were confirmed by pathology report	Area-level measure using residential address and census data to calculate composite index scores of neighbourhood disadvantage and affluence	Age, race	Selection bias	Lower level of affluence associated with later stage at diagnosis. No association between disadvantage and stage at diagnosis.	Difference in prevalance between higher and lower affluence= −0.15 (−0.26, −0.05)	0.004 (affluence)
										Difference in prevalance between higher and lower disadvatnage = 0.10 (−0.06, 0.24)	0.2 (disadvantage)
Lyratzopoulos et al. [9]	England	2006–2010	Case series	2744	Cancer registry: ICD-10 code C56	Area-level measure - Index of Multiple Deprivation	Tumour type, age	Stage missing for 11% of cohort	No evidence of a difference in stage at diagnosis according to deprivation	0.74 (0.52–1.03) most deprived vs. most affluent	0.077
Brewster et al. [11]	Scotland	1992–1994	Case series	1387	Cancer registry (presumed C56, which would include borderline and non-epithelial tumours)	Not clear, stratified into affluent, middle and deprived	None - univariate analysis	Not clear how SES measured. Large proportion of missing data (8.7%)	Borderline significance. Possibility that deprived patients have more advanced disease.	not reported	0.06
Morris, Sands and Smith [10]	USA	1996–2006	Case series	16,228	Epithelial ovarian cancers (including borderline tumours) from the California Cancer Registry	Area-level index score of deprivation calculated using 7 indicators of SES	Age, race, insurance status	All Stage II cancers excluded	No evidence of an association between SES and diagnosis of early (stage I) vs. late (stage III and IV) disease.	1.1 (0.97–1.21)	0.142
Praestegaard et al. [12]	International (pooled analysis of 18 studies)	1989–2010	Pooled analysis of cases from case-control studies	10,601	Primary invasive epithelial OC	Education level (self-reported)	BMI, smoking, ethnicity	No adjustment for comorbidity	Small increased risk of being diagnosed with advance stage disease if less than high school education attained.	1.15 (1.03−1.28) (pooled ORs)	<0.05
Ibfelt et al. [13]	Denmark	2005–2010	Case series	2873	Borderline tumours included. Data from Danish Gynaecological Cancer Database (data entered by gynaecologists).	Education level, amount of disposable income, cohabitation status (all from national registers)	Charlson comorbidity index, BMI, smoking status, ASA score (Amercian Society of Anaesthaesiologists)		Level of disposable income associated with stage at diagnosis (*p* = 0.04), length of time spent in education weakly associated (*p* = 0.07).	0.83 (0.70−0.98), middle vs. highest quartiles; 0.86 (0.70−1.07), lowest vs. highest quartiles	0.04 (income)
										1.25 (1.12–1.40), higher vs medium education **; 1.12 (0.88–1.41), higher vs short education **	0.07 (education)

* nominal statistical significance, exact *p*-value not provided. ** ‘short’ education defined as 7 or 9 years; ‘medium’ defined as 8 or 10–12 years; ‘higher’ defined as ≥12 years.

**Table 2:** Complementary ordinal logistic regression—reduced sample size to include follow-up questionnaire data.

	OR	95% Confidence Intervals		*p*-Value
IMD *	1.34	0.99	1.80	0.054
**Ever smoker**	1.56	0.95	2.55	0.077
**Educational attainment**				
**None**	referent			
**‘O’ level (approx 16 years old)**	1.12	0.50	2.50	0.790
**‘A’ level, clerical or commercial qualification (approx. 18 years old)**	1.02	0.55	1.92	0.942
**Nursing, teaching or University qualification (18+ years old)**	1.74	0.89	3.40	0.103
**Alcohol consumption (units/week)**				
**0**	referent			
**<** **6**	0.90	0.48	1.68	0.730
**7–15**	0.70	0.32	1.52	0.364
** ≥ ** **16**	0.32	0.11	0.93	0.036
**BMI (kg/(M^2^))**				
**Underweight (<18.5)**	0.39	0.25	1.03	0.062
**Normal (≥18.5 and <25)**	referent			
**Overweight (≥25 and <30)**	0.95	0.56	1.64	0.470
**Obese class I (≥30 and <35)**	0.51	0.25	1.03	0.062
**Obese class II (≥35 and <40)**	0.31	0.06	1.51	0.147
**Obese class III (≥40)**	7.23	0.62	84.31	0.114
**Age at diagnosis**	1.05	1.01	1.09	0.006
**Stage at diagnosis**				
**I vs II,III,IV**	1.60	−1.03	4.23	
**I,II vs III IV**	2.20	−0.43	4.83	
**I,II,III vs IV**	4.98	2.28	7.68	

* IMD: Index of Multiple Deprivation.

**Table 3:** Baseline characteristics of the study cohor

	All		I		II		III		IV	
	*n*	%	*n*	%	*n*	%	*n*	%	*n*	%
**All eligible Women**	**457**	**100**	**66**	**14.44**	**39**	**8.53**	**265**	**57.99**	**87**	**19.04**
**Covariate**										
**Cancer Type**										
I	62	13.57	40	60.61	9	23.08	10	3.77	3	3.45
II	345	75.49	25	37.88	29	74.36	223	84.15	68	78.16
Type uncertain	50	10.94	1	1.52	1	2.56	32	12.08	16	18.39
**OCP Ever Use**										
Yes	229	50.11	31	46.97	21	53.85	139	52.45	38	43.68
No	228	49.89	35	53.03	18	46.15	126	47.55	49	56.32
**Smoking**										
Yes	125	27.35	20	30.30	8	20.51	70	26.42	27	31.03
No	150	32.82	25	37.88	16	41.03	85	32.08	24	27.59
Missing	182	39.82	21	31.82	15	38.46	110	41.51	36	41.38
**Alcohol Consumption (units/week)**										
0	68	14.88	11	16.67	3	7.69	41	15.47	13	14.94
<1–6	144	31.51	23	34.85	12	30.77	81	30.57	28	32.18
7–15	50	10.94	8	12.12	6	15.38	25	9.43	11	12.64
≥16	15	3.28	3	4.55	3	7.69	9	3.40	0	0.00
Missing	180	39.39	21	31.82	15	38.46	109	41.13	35	40.23
**Educational Attainment**										
None	92	20.13	13	19.70	7	17.95	56	21.13	16	18.39
‘O’ level (approx. 16 years old)	37	8.10	6	9.09	4	10.26	20	7.55	7	8.05
‘A’ level, clerical or commercial qualification (approx. 18 years old)	71	15.54	10	15.15	8	20.51	45	16.98	8	9.20
Nursing, teaching or University qualification (18+ years old)	68	14.88	13	19.70	5	12.82	29	10.94	21	24.14
Missing	189	41.36	24	36.36	15	38.46	115	43.40	35	40.23
**BMI (kg/(M^2^))**										
Underweight (<18.5)	3	0.66	0	0.00	1	2.56	2	0.75	0	0
Normal (≥18.5 and <25)	206	45.08	21	31.82	21	53.85	132	49.81	32	36.78
Overweight (≥25 and <30)	155	33.92	27	40.91	9	23.08	81	30.57	38	43.68
Obese class I (≥30 and <35)	67	14.66	12	18.18	6	15.38	37	13.96	12	13.79
Obese class II (≥35 and <40)	20	4.38	6	9.09	2	5.13	10	3.77	2	2.30
Obese class III (≥40)	6	1.31	0	0.00	0	0.00	3	1.13	3	3.45
**Tubal Ligation**	84	18.38	9	13.64	2	5.13	56	21.13	17	20.69
**Hysterectomy**	89	19.47	14	21.21	11	28.21	48	18.11	16	18.39
**Ethnicity**										
White	446	97.59	66	100	38	97.44	255	96.23	87	100.00
Black	2	0.44	0	0	0	0.00	2	0.75	0	0.00
South Asian	3	0.66	0	0	0	0.00	3	1.13	0	0.00
Chinese	1	0.22	0	0	1	2.56	0	0.00	0	0.00
Other	4	0.88	0	0	0	0.00	4	1.51	0	0.00
Missing	1	0.22	0	0	0	0.00	1	0.38	0	0.00
	**Median**	**IQR**	**Median**	**IQR**	**Median**	**IQR**	**Median**	**IQR**	**Median**	**IQR**
**Age at diagnosis**	68.49	63.63–73.17	67.49	60.30–72.64	66.38	62.31–69.44	69.16	64.25–73.17	69.89	65.1–74.32
**Duration of OCP use (years)**	4	2–9	5.5	2.75–10	6	2–11	3.5	2–8	3	2–6.5
**Number of pregnancies <6 months**	0	0–1	0	0–0	0	0–0	0	0–1	0	0–1
**Number of pregnancies ≥6 months**	2	2–3	2	1–3	2	1–3	2	2–3	2	2–3

**Table 4:** Ordinal logistic regression of socioeconomic status and OC stage at diagnosis.

	OR	95% Confidence Interval		*p*-Value
IMD *	1.29	1.05	1.59	0.02
**BMI (kg/(M^2^))**				
Underweight (<18.5)	0.39	0.06	2.60	0.33
Normal (≥18.5 and <25)	referent			
Overweight (≥25 and <30)	1.06	0.70	1.62	0.78
Obese class I (≥30 and <35)	0.80	0.47	1.38	0.43
Obese class II (≥35 and <40)	0.48	0.20	1.17	0.11
Obese class III (≥40)	5.48	1.13	26.53	0.04
**Age at diagnosis**	1.05	1.02	1.07	0.002
**Stage at diagnosis**				
I vs II,III,IV	1.10	−0.82	3.01	
I,II vs III,IV	1.69	−0.23	3.60	
I,II,III vs IV	4.46	2.50	6.43	

* IMD: Index of multiple deprivation.
